# Comprehensive Study on the Performance of Waste HDPE and LDPE Modified Asphalt Binders for Construction of Asphalt Pavements Application

**DOI:** 10.3390/polym14173673

**Published:** 2022-09-04

**Authors:** Usman Ghani, Bakht Zamin, Muhammad Tariq Bashir, Mahmood Ahmad, Mohanad Muayad Sabri Sabri, Suraparb Keawsawasvong

**Affiliations:** 1 Department of Engineering, Ed.8, University of Palermo, Viale Delle Scienze, 90128 Palermo, Italy; 2 Department of Civil Engineering, CECOS University of IT & Emerging Sciences, Peshawar 25000, Pakistan; 3 Department of Civil Engineering, University of Engineering and Technology Peshawar (Bannu Campus), Bannu 28100, Pakistan; 4 Peter the Great St. Petersburg Polytechnic University, 195251 St. Petersburg, Russia; 5 Department of Civil Engineering, Thammasat School of Engineering, Thammasat University, Pathumthani 12120, Thailand

**Keywords:** waste polyethylene, spectroscopic analysis, morphological analysis, XRD, creep analysis, SEM

## Abstract

This research is aimed at investigating the mechanical behavior of the bitumen by the addition of high-density polyethylene (HDPE) and low-density polyethylene (LDPE) obtained from waste plastic bottles and bags. Polymers (HDPE and LDPE) with percentages of 0%, 2%, 4%, and 6% in shredded form by weight of bitumen were used to evaluate the spectroscopic, structural, morphological, and rheological properties of polymer-modified binders. The rheological properties for different factors; viscosity (ἠ) from Rotational Viscometer (RV), rutting factor G*/Sin (δ), fatigue characteristics G*. Sin (δ), for the modified binder from dynamic shear rheometer (DSR), Short and long-term aging from rolling thin film oven (RTFO), and pressure aging vessel (PAV) was determined. The thermal characteristics, grain size, and texture of polymers for both LDPE and HDPE were found using bending beam rheometer (BBR) and X-ray diffraction (XRD), respectively. Fourier transform infrared (FTIR) analysis revealed the presence of polymer contents in the modified binder. Scanning electron microscopy (SEM) images revealed the presence of HDPE and LDPE particles on the surface of the binder. Creep Rate (m) and Stiffness (S) analysis in relationship with temperature showed a deduction in stress rate relaxation. Results have revealed the best rutting resistance for 6% HDPE. It also showed an improvement of 95.27% in G*/Sin (δ) which increased the performance of the bituminous mix. Similarly, the addition of 4% LDPE resulted in maximum dynamic viscosity irrespective of the temperatures. Moreover, fatigue resistance has shown a significant change with the HDPE and LDPE. The festinating features of waste plastic modified binder make it important to be used in the new construction of roads to address the high viscosity and mixing problems produced by plastic waste and to improve the performance of flexible pavements all over the world.

## 1. Introduction

The heavy traffic and loading over time adversely affect the rheological performance of the flexible pavements. The upper part of the flexible pavements is mainly composed of bitumen, aggregates, and filler materials to bear the stresses imposed by adverse traffic conditions. Bitumen as a binding material is made up of hydrocarbons and has a strong influence on the performance of asphalt pavements [1]. 

The rheological properties of bitumen can enhance the structural and functional performance of asphalt pavements [2]. Material characterization is one of the major factors affecting pavement design. Supposing the materials are unable to provide better resistance to fatigue and permanent deformation; in this case, distress may likely occur in asphalt pavements, which may affect the structural and functional properties of asphalt pavements. When the performance of the pavement reaches a level where the desired function of the pavement is no longer available, or the pavement is not optimally providing, the desired service is termed as failure. In flexible pavements, three major types of distress cause failure in the structure, i.e., rutting, fatigue cracking, and to an extent, thermal cracking [3]. For the last 40 years, researchers have been putting their efforts to modify bitumen by employing the addition of polymers to enhance its physical and rheological properties [4]. For developing countries such as Pakistan, the addition of waste plastic in bituminous materials is one of the easiest available resources to strengthen the bitumen and reduce the environmental hazards produced by these wastes. According to the Environmental Protection Agency (EPA), Pakistan has produced 3.9 million tonnes of plastic waste during the year 2020, in which 70 percent (2.6 million tonnes) was mismanaged. According to the EPA, this amount of plastic waste may be tripled by 2040 [5]. 

The modified bitumen obtained from waste plastic is used as either elastomers or plastomers [6,7]. The rheological properties and viscosity function can be highly influenced by the addition of elastomers and plastomers from 2% to 6% by the weight of bitumen [8]. The process of adding bitumen with different polymers is termed a wet process [9]. Depending on the nature, size, the type of equipment used for mixing, and the shape of polymers [10], several properties such as resistance to permanent deformation, resistance to moisture-related distresses, fatigue life, and the achievement of high stiffness at a high temperature, can be improved by adding waste plastics [11,12,13,14]. By substituting the HDPE and LDPE, the thinner pavement cross-sections can be developed more efficiently due to their mechanical strength and chemical compatibility with the original binder [15]. 

The addition of nano-silica from 1% to 6% by weight of bitumen in the polymer-modified binder has improved its viscoelastic properties and the rutting factor G*/Sin (δ) and fatigue characteristics G*.Sin (δ) [16]. Styrene–butadiene–styrene (SBS) polymer modified asphalt mixtures and analysis of strain distribution showed that SBS polymer modified bitumen is improving the stress levels within the asphalt mastic [17,18,19,20]. The low percentage of SBS revealed the dispersion of polymers particles in a continuous phase of bitumen, as the original binder has swollen the small polymer’s globules. As a result, compatible fractions are spread in the homogenous form in a continuous phase of bitumen [17]. In another investigation of adding 2% to 6% nano-silica in polymer-modified bitumen, results showed a delay in the aging of bitumen. The rutting and fatigue parameters were improved along with the viscoelastic properties of a polymer-modified binder [21,22]. Aging and regeneration can change the microstructural performance and morphological performance of bitumen [23,24,25]. The microstructures investigations of SBS polymer modified binder in addition to Montmorillonite (MMT) using X-ray diffraction (XRD) and FTIR indicated that the softening point and aging index have decreased due to the addition of Na+. The introduction of Na+ in SBS polymer-modified binder has created a phase-separated structure [26]. The surface properties of polymer-modified bitumen were determined using scanning electron microscopy and AT-FTIR. The contact angle showed a decrease from 107.7° to 4.7° while the oxygen atomic percentage was increased from 7.12% to 13.15%, respectively. This exhibits the chemical interaction of the polymer-modified binders. Chemical capabilities in terms of oxidation in aged bitumen can be seen without the evolution of interactions [27]. 

Although the addition of nano-silica and SBS has its advantages in polymer modification there is still a lack of research on the affinity of polymers with bitumen. At a low strain level, there is no slippage of polymer chains or change in morphological properties but at a higher strain and higher cyclic loads, the research question still remains uncertain [28].

Another study for investigating the physical and rheological properties of asphalt binders was done by introducing the nanoparticles of aluminum oxide (Al_2_O_3_) by weight of 3%, 5%, and 7% bitumen. The addition of 5% aluminum oxide by weight of bitumen, reduced the phase angle (δ), and the complex shear modulus (G*) was increased, respectively [29]. However, the bond between nanoparticles and bitumen still needs further investigation in terms of morphological analysis. 

Although extensive research has been conducted so far on polymer-modified bitumen, there is a need for improvement in the polymer in terms of its rheology, morphology, and creep assessment [30]. Recently, researchers have reported some critical issues with using polymers as modifiers utilizing the wet process. These issues include the mixing problems related to high viscosity at the higher temperature, the affinity of polymers with bitumen, and the high cost of its modification [9]. Due to the ample amount of plastic waste available in Pakistan, this research uses two waste plastics, i.e., HDPE obtained from waste bottles and LDPE obtained from waste plastic bags in shredded form, intending to investigate the above critical issues. 

**Figure 1:**
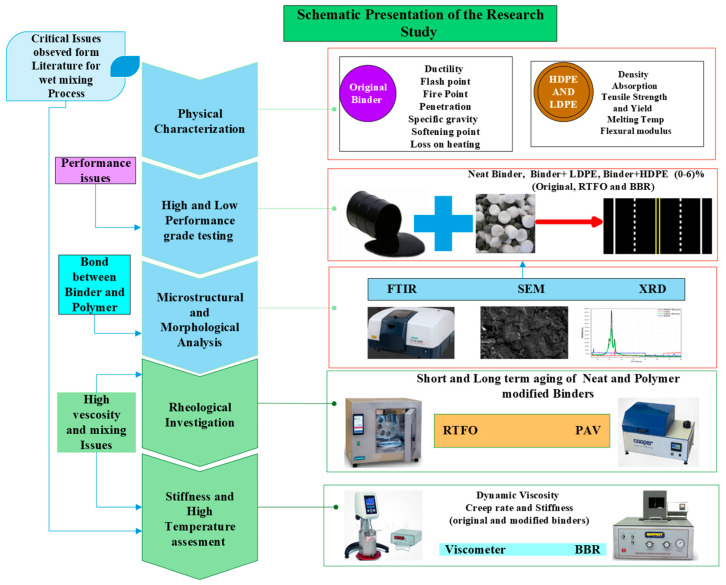
A schematic presentation of the critical issues observed in the current literature.

The morphological properties of neat binders have been compared with HDPE and LDPE modified binders in order to predict the bond linkage between neat binders and polymers. The performance of a neat and modified binder has been investigated in the Section 2.4.2 rheological investigation section. Dynamic Viscosity measurements have discussed the high viscosity and mixing issues of adding polymers during high temperatures. The comparison of the stiffness for neat and modified binders has been discussed in creep analysis. The schematic representation of the study has been shown in Figure 1. 

## 2. Materials and Methods

### 2.1. Materials

The fundamental materials used in this research work were bitumen, high-density polyethylene (HDPE), and low-density polyethylene (LDPE) obtained from a local depository in Peshawar Pakistan. The waste plastic bottles and bags were first washed, dried, and shredded to pass through sieve No.4 [30]. The performance and penetration grade of bitumen was PG 58-22 and grade 60/70, respectively, obtained from Attock Oil Refinery Limited (ARL), Pakistan. Table 1 represents the physical properties of the asphalt binder, while the physical properties of HDPE and LDPE used in this investigation are presented in Table 2. 

### 2.2. Performance Grade Testing 

Performance grade (PG) of conventional and HDPE and LDPE modified bitumen was determined by following AASHTO M 320 specifications, as is presented in Table 3. 

### 2.3. Preparation of PE Modified Bitumen

The HDPE and LDPE were mixed separately with bitumen in an agitator at 3000 rpm after applying the heat treatment (163 °C for 1 h in an oven). The HDPE and LDPE were mixed separately with different percentages of 2%, 4%, and 6% by weight of bitumen. The mixture was reheated at 163 °C after the addition of the modifier for 10 min.

### 2.4. Testing Procedures

#### 2.4.1. SEM, FTIR, and XRD Analysis

As the chemistry of the polymers is quite different from that of bitumen, the interaction of HDPE and LDPE with bitumen was determined using different characterization tools. The FTIR spectroscopy was done to identify the functional group in neat and polymer-modified binders. The specifications followed for high- and low-density polyethylene were AASHTO T 302–15. The chemical bonding of HDPE and LDPE with bitumen was investigated by FTIR (Perkin Elmer L1600). This test was performed in the wavenumber range (4000–400) cm^−1^ by using the KBr technique. For performing the FTIR, the testing temperature was 25 degrees Celsius. The bond concentration or the identification of the functional group can be identified by the intensity of the peak spectra in FTIR [31]. The polymer’s interaction with the neat bitumen enhances the morphological properties of the HDPE and LDPE modified binder and needs to be understood using scanning electron microscopy (SEM-JEOL JSM 5910).

The XRD analysis of neat bitumen and modified binder has been studied to examine their structural features. The crystalline features of the materials were studied by using a dedicated software, Bruker AXS, D8 advance. X-ray diffraction (XRD) was used to investigate the diffraction patterns, grain size, and texture of the modified bitumen. The X-Ray Diffraction, abbreviated commonly as XRD, is a non-destructive research tool used for the study of crystalline material structure. It studies the structure of the crystal used to classify the crystalline phases contained in a substance and thus discloses information about the chemical composition of the material [32].

#### 2.4.2. Rheological Investigation

**Figure 2:**
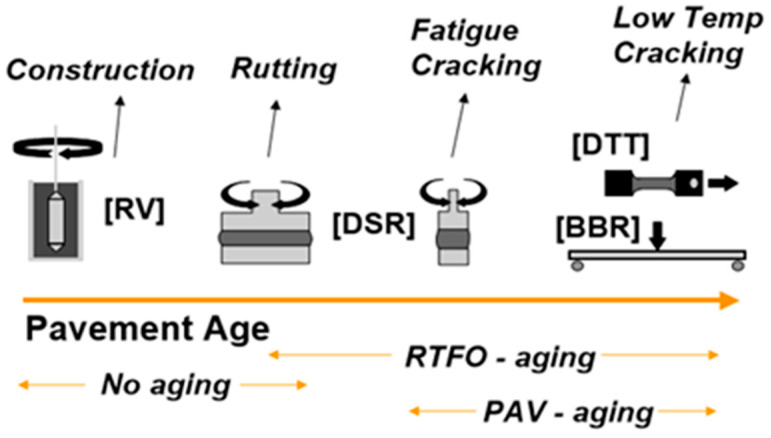
The schematics of rheological investigations of neat and modified bitumen.

The rheological assessments were carried out to determine the viscosities, rutting resistance along with short- and long-term aging of HDPE and LDPE modified asphalt binders using the dynamic shear rheometer (DSR AASHTO T 315). All tests were performed at different temperatures (58 °C, 64 °C, 70 °C, and 76 °C) following the specifications of (AASHTO T 240-09) for short-term aging RTFO-aged and (ASTM D 6521) for long-term aging PAV-aged, the rutting factor G*/Sin (δ) and fatigue characteristics G*Sin (δ) of original and polymers modified binders were determined. The frequency of testing samples was 10 rad/s according to the standard specifications. According to the (AASHTO T-315), the temperature was maintained at 58 °C, 64 °C, 70 °C, and 76 °C for rutting resistance determination. The original samples for G*/Sin (δ) were tested from a minimum value of 1.0 KPa and RTFO aged binder samples at a minimum value of 2.2 KPa at maximum temperature. However, the minimum value of G*. Sin (δ) was 5000 KPa. Schematics of rheological investigations of neat and modified bitumen are depicted in Figure 2 [33,34,35].

#### 2.4.3. Short-Term and Long-Term Aging of a Binder

The Rolling Thin Film Oven Test (RTFO) was performed to discover the short-term aging of bitumen according to the standard specifications of AASHTO T-240. The total duration of the test was 85 min and the temperature was 163 °C in the RTFO bottle [34]. The volatile particles involved in bitumen cause short-term aging. For long-term aging, the sample was put into the plates and Pressure Aging Vessel (PAV) for 20 h. The PAV can predict the binder up to 10 years of its service life. Further physical testing was performed by storing the binder in cans.

#### 2.4.4. Dynamic Viscosity Test 

The rotational viscometer (RVDV-111) was used to find out the dynamic viscosity of original and modified samples. The percentages of polymer-modified bitumen were from 2% to 6% by weight of bitumen. According to the specifications of AASHTO T-316, the high temperature for the viscosity test was from 135 °C to 165 °C. The changing interval of temperature was 10 °C, respectively. The rotational speed of the cylindrical spindle was 20 rpm [36].

#### 2.4.5. Bending Beam Rheometer Tests for Creep

**Figure 3:**
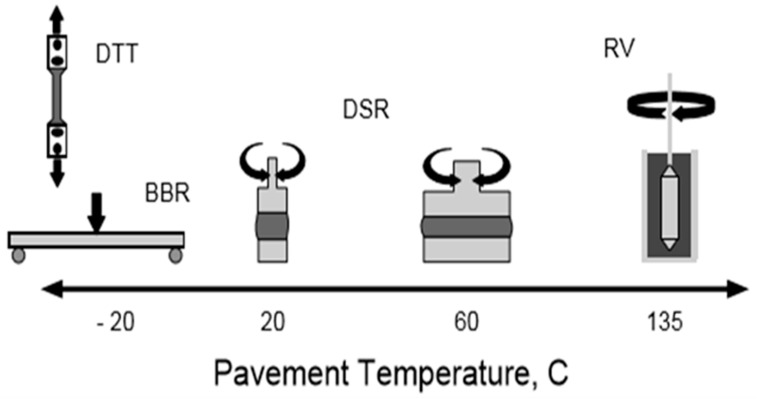
The schematics of creep stiffness (S) and creep rate (m) analysis.

The low-temperature cracking or thermal cracking was measured by BBR. According to the specifications of AASHTO T-313 at three different temperatures of 0 °C, −6 °C, and −12 °C, the BBR test was performed. The length, thickness, and width of the beams of bitumen were 127 mm, 6.4 mm, and 12.7 mm, respectively. The temperature was constantly maintained for 60 min. After the preloading conditions, a 100 g load was applied to the rectangular beam at a constant rate for measuring the deflection at the center [37]. The loading time was from 8 to 240 s to discover the creep stiffness (S) and creep rate (m). Schematics for creep rate (m) and creep stiffness (S) are shown in Figure 3.

## 3. Results and Discussions 

### 3.1. Scanning Electron Microscopy

**Figure 4:**
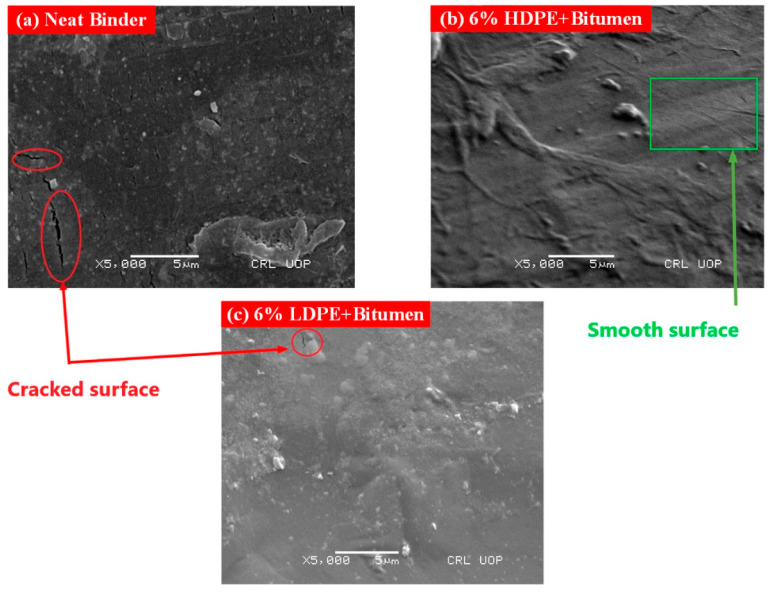
SEM images: (**a**) Neat Binder; (**b**) HDPE modified bitumen; (**c**) LDPE modified bitumen.

The scanning electron microscopy (SEM) of controlled and HDPE and LDPE modified bitumen is shown in Figure 4. The micro-cracks that appeared on the surface of a neat binder can be seen in Figure 4a. This crack growth may be increased under the cyclic loading imposed by the heavy traffic and will cause severe microstructural disorder in flexible pavements [25]. As the molecular chains of the 6%, HDPE interacts with the neat binder as shown in Figure 4b; the surface of the modified bitumen became smooth and showed no appearance of micro-cracks on the surface. The continuous matrix of polymers, with the addition of 6% by weight of bitumen, is clearly visible in Figure 4b. The packed surface of HDPE-modified bitumen without micro-cracks may provide better resistance against rutting and fatigue characteristics caused by the heavy loading in flexible pavements. As depicted in Figure 4c, some of the micro-cracks still appeared with the same amount of LDPE (6%) added by the weight of bitumen. With the addition of 6%, LDPE the discontinuous matrix of LDPE is observed. Similarly, phase dispersion of bitumen can be seen when the percentage of LDPE is greater than 6%. 

### 3.2. Fourier Transform Infrared (FTIR) Analysis

**Figure 5:**
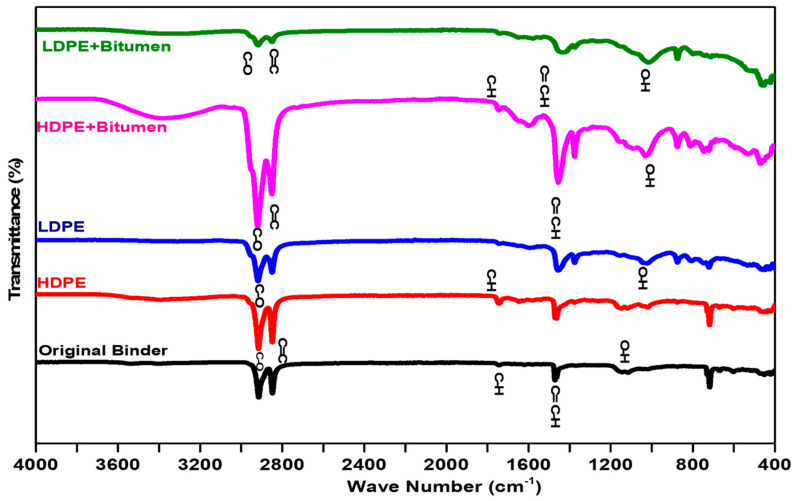
An FTIR analysis of neat, HDPE, and LDPE modified bitumen.

The FTIR analysis for neat bitumen, HDPE, and LDPE modified bitumen is presented in Figure 5 below. The HDPE and LDPE modified bitumen showed the emergence of peaks and the presence of OH groups shown in Figure 5. Other peaks developed were due to the absorption of HDPE and LDPE in bitumen. The overtones around 2000–1800 cm^−1^ can be clearly seen in Figure 5, which confirms the presence of aromatic carbons. The C-H stretching of organic compounds represented in the range of the peaks in Figure 5 is between 2850–3000 cm^−1^ [32,33,34,35,36,37,38]. Values for the other peaks in Figure 5 are between 3000 cm^−1^ and 1500 cm^−1^, proving the presence of polar groups. The polar group is relatively responsible for creating a stronger bond between a neat binder and dispersed polymer [39]. The bending of the C-H group can be seen at 1570 cm^−1^. It can also be noted in the figure that the neat HDPE act as apolar or production of alkene or olefin with a highly crystalline structure. An alkene or olefin is a type of unsaturated molecule that contains one carbon to carbon double bond. As compared to the HDPE, the LDPE is less apolar and has a less crystalline structure. Due to the high crystallinity found inside the polymers, there will be a sufficient amount of improvement in the blended mix of polymers with neat bitumen [40]. 

### 3.3. X-Rays Diffraction (XRD) Analysis 

**Figure 6:**
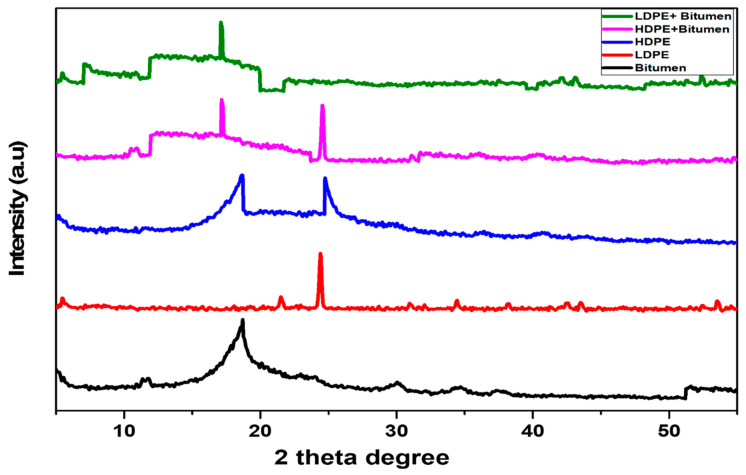
An XRD analysis of neat, HDPE, and LDPE modified bitumen.

As can be seen in Figure 6, the black line represents the neat bitumen that is not crystallized. The peak 2θ degree for HDPE in addition to bitumen was 26.71° on a scale of 0 to 60 with a scan speed of 2 deg/min with an intensity of 5000 (a.u). The LDPE in addition to bitumen 25.20° with an intensity of 4189 (a.u). As can be seen from Figure 6, at the lower angle the modified bitumen shows a semi-crystalline phase. As the percentage of absorption increases the crystalline phase also increases. Previous research has reported the crystalline behavior of LDPE at 4% by weight of bitumen showed a significant decrease in permanent deformation, fatigue, and thermal crack resistance at high, intermediate, and low temperatures [41]. Another research confirmed that the highly crystalline bitumen-modified mixture can improve the thermal stability of the mixture which has a direct effect on the rheological properties of the asphalt mixture [42]. The degree of crystallinity for HDPE is more as compared to the LDPE but at a higher temperature in the wet mixing process, the HDPE has a high viscosity and mixing issues with the neat binder. From the conducted XRD on neat and modified bitumen, it can be concluded that the crystalline structure of the polymers and their modification enhance a key characterization of the chemical properties of the modified bitumen. Through the addition of polymers, there is an improvement in the elastic properties of a modified binder. As a result, it will provide better resistance to permanent deformation. 

## 4. Rheological Performance Analysis 

**Figure 7:**
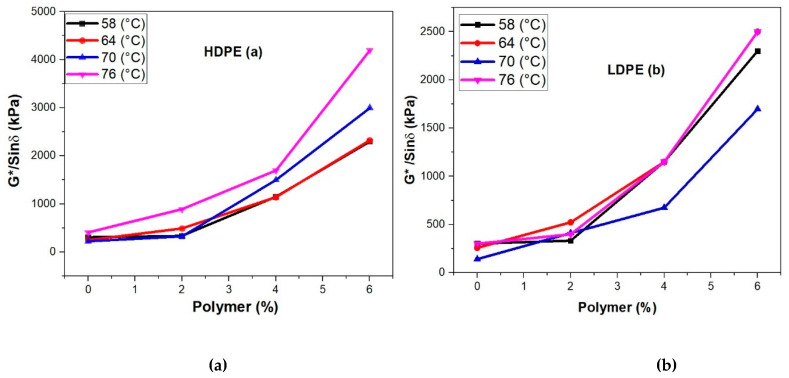
(**a**) A comparison between HDPE modified bitumen and G*/Sin (δ) in unaged bi tumen, (**b**) comparison between LDPE modified bitumen and G*/Sin (δ) in unaged bitumen.

From 2% to 6% addition of HDPE and LDPE, the rheological performance of neat binder, i.e., PG 58–22 has been compared. Figure 7a,b depicted the rheological performance of the unaged binder. The temperatures 58, 64, 70, and 76 °C were used for finding the rutting factor i.e., G*/Sin (δ). The addition of HDPE and LDPE depicted the increase in G*/Sin (δ). As a result, the rutting factor was increased with the addition of 2%, 4%, and 6% HDPE and LDPE. The phase angle (θ) for the temperature of 58, 64, 70, and 76 °C was determined using DSR.

**Figure 8:**
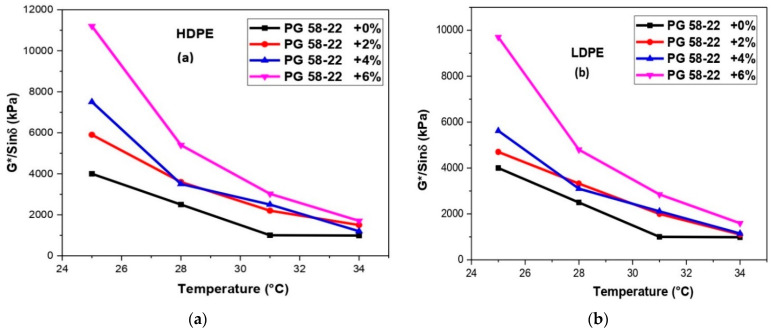
(**a**) An analysis of G*/Sin (δ) and temperature with HDPE modified bitumen for long-term aging, (**b**) an analysis of G*/Sin (δ) and temperature with LDPE modified bitumen for long-term aging.

The rutting resistance as compared to the neat binder was increased with the addition of HDPE and LDPE. High values of rutting parameters, i.e., G*/Sin (δ) may decrease the chances of rutting in asphalt, also summarized by previous research [43,44]. As compared to the original binder after the addition of polymers in Figure 7a, the values of 480, 940, and 1970 Pa showed 13.8%, 26.8%, and 56.2% improvement in rutting resistance. The temperature was maintained at 64 °C for adding HDPE from 2 to 6% in the bitumen. Figure 7b depicts the results of LDPE, and it can be noted that the values 500, 1100, and 2250 Pa showed 19.23%, 42.3%, and 86.5% improvement in rutting resistance. After conducting the short-term aging test, Table 4 shows the values obtained from RTFO residues reused in DSR for different phase angles. As the percentage of polymers increases the values of phase angle decrease. Moreover, after the conduction of the Long-term aging test, Table 5 showed the values obtained from PAV residues reused in DSR for different phase angles at 25 °C. Figure 8a,b depicted the results for long-term aging after the addition of HDPE and LDPE. It can also be noted that according to Table 5 the percentage of polymers increases the phase angle decreases, which shows an increase in elastic properties of the bitumen.

The phase angle of pure polymer content is less than the neat binder, irrespective of the temperature conditions. Irrespective of the temperature change, the reduction of phase angle is due to the rich phase of the polymer that behaves like an elastic filler in a neat binder matrix [45]. This shows that the polymer’s addition improves the high-temperature performance of asphalt mortar. A decrease in phase angle (θ) was observed with the usage of the polymers, as the phase angle is acting as a lag between the applied shear stress and resulting in the shear strain. The lesser the phase angle, the more asphalt mortar can resist permanent deformation, as shown in Table 4 and Table 5, respectively.

## 5. Dynamic Viscosity Analysis

**Figure 9:**
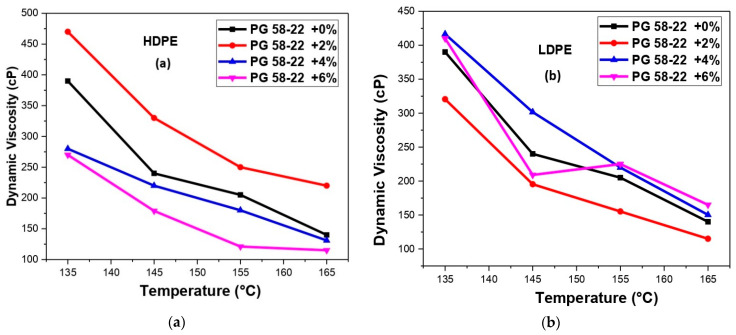
(**a**) The relation of dynamic viscosity with temperature for HDPE modified bitumen, (**b**) relation of dynamic viscosity with temperature for LDPE modified bitumen.

The results of viscosity for HDPE and LDPE are shown in Figure 9a,b, respectively. The increase in the viscosity values showed a marked difference with the addition of polymers. The maximum dynamic viscosity is obtained by adding 2% of HDPE at all the temperatures, as shown in Figure 9a. The percentage of Dynamic viscosity increases at a temperature of 135, 145 155, and 165 °C was 9.67%, 15.8%, 25%, and 18.1%, respectively. The maximum dynamic viscosity is obtained by adding 4% of LDPE at all the temperatures, as shown in Figure 9b. The percentage of Dynamic viscosity increases at temperatures of 135.0, 145.0, 155.0 and 165.0 °C was 5.90%, 21.80%, 5.0% and 18.10%, respectively [36]. 

## 6. Creep Rate and Creep Stiffness Analysis

**Figure 10:**
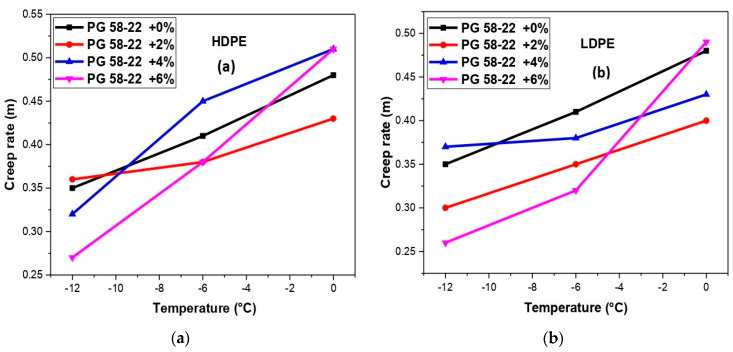
(**a**) An analysis of creep rate (m) and temperature with HDPE-modified bitumen, (**b**) analysis of creep rate (m) and temperature with LDPE modified bitumen.

**Figure 11:**
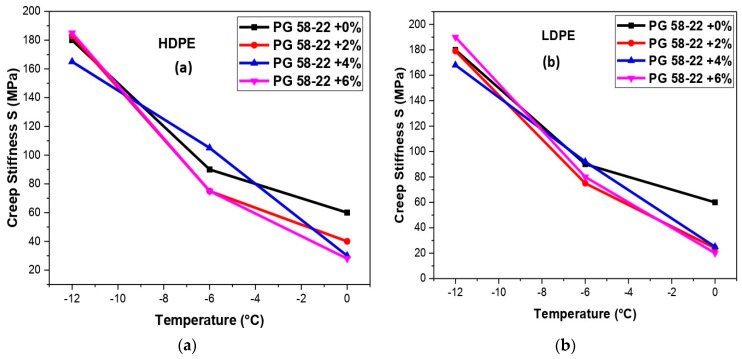
(**a**) An analysis of creep stiffness (S) and temperature with HDPE-modified bitumen, (**b**) analysis of creep stiffness (S) and temperature with LDPE-modified bitumen.

By applying the loading time of 60 s for HDPE and LDPE modified bitumen, creep rate (m) and creep stiffness (S) were found using the software as depicted in Figure 10 and Figure 11. The range for (m) should be greater than 0.300 while the (S) should below 300 MPa [46]. The relation between temperature and creep rate (m) for HDPE and LDPE can be seen in Figure 10a,b. The trend of the graph was decreasing at −6 °C for LDPE, but the HDPE was according to the desired criterion.

Figure 11a,b represented the relationship between S and temperature for HDPE and LDPE, respectively. The polymer-modified bitumen becomes stiffer at low temperatures. At low temperatures, adding both HDPE and LDPE at 6% has shown higher creep stiffness values than the original binder, i.e., 185 MPa [47]. The values of S have a relationship with thermal stress, which is developed in pavements that undergo shrinkage. Hence, there could be thermal cracking in the pavement structure. The stress rate has a relationship with the (m) values; whenever it decreases, the relaxation in the stress rate also decreases. So, it can be concluded that for long-term pavement performance Low (S) values and Higher (m) values are required.

## 7. Summarized Conclusions

This research was conducted to assess the morphological, rheological and dynamic viscosity, and creep characteristics of waste HDPE and LDPE modified asphalt binders. The major conclusions drawn from this investigation are summarized as follows:

- This study concluded that the induction of both the HDPE and LDPE decreased the phase angle (θ) which shows that the asphalt mortar is more resistant to permanent deformation. The high-temperature viscosity of the asphalt binder increased, and a marginal increase was noticed when 2.0% HDPE, and 4.0% LDPE were used in the mix.
- The decreased viscosity facilitates the bituminous mix during mixing and compaction. This could be due to a decrease in sphere particles, which gives a micro bearing effect due to the reduction of sphere particles. On the other hand, the effect of spherical particles on the rutting parameter showed that the greater the rutting parameter would be the rutting susceptibility. The 4.0% LDPE with greater sphere particles and less viscosity than HDPE can be utilized for road construction.
- The addition of 2% HDPE and 4% LDPE by weight of bitumen showed improvement in rutting resistance of 13.8% and 42.3%, respectively. These percentages also reported the maximum dynamic viscosity measurements, i.e., 21.80% at 145 °C. However, increasing the percentage of polymers can result in high viscosity and may have a mixing issue on plant-level modified bitumen preparation.
- The trend of the graph for creep rate (m) was decreasing at −6 °C for LDPE but the trend of HDPE was according to the desired criterion. For the modified bitumen, the Creep stress (S) and its relationship with the temperature showed a decreasing trend, which shows relaxation in stress rate, also decreases.
- The FTIR peaks showed the addition of HDPE and LDPE. The polymers modified bitumen by HDPE and LDPE showed the emergence of peaks. As compared to the HDPE, the LDPE is less apolar and has a less crystalline structure. Due to the high crystallinity found inside the polymers, there will be a sufficient amount of improvement in the blended mix of polymers with neat bitumen.
- By the addition of 6% LDPE, the discontinuous matrix of LDPE is observed. However, it can be concluded that by increasing the percentage of LDPE from 6% the morphological improvement, along with phase dispersion of bitumen can be observed.
- By the X-Rays diffraction, the degree of crystallinity for HDPE is more as compared to the LDPE but at a higher temperature in the wet mixing process the HDPE has a high viscosity and the mixing issues with the neat binder.
- The above study recommended the use of waste plastics obtained from waste plastic bottles and bags for the under-construction asphalt pavements in order to improve the bearing capacity of the wearing surface, especially the under-construction projects all over the world, like the China–Pakistan Economic Corridor (CPEC).
- For the high viscosity issues and mixing problem using a wet mixing process, this research concluded that at a high temperature the waste LDPE proved to be the better modifier as compared to the HDPE, but there is a still a need for further studies on microstructural, morphological and dynamic viscosity of HDPE and LDPE modified binders.

## Figures and Tables

**Table 1:** The physical properties of bitumen PG 58-22.

Test Type	Standard	Result
Ductility @ 25 °C,	ASTM D 113	75
Flash point Test (°C)	AASHTO T 48-96	250
Fire Point Test (°C)	AASHTO T 48-96	271
Penetration @ 25 °C	ASTM D 5-97	67
Specific gravity (g/cm^3^)	AASHTO T 228	1.017
Softening point (°C)	ASTM D 36-95	43
Loss on heating (%)	AASHTO T 47	0.011

**Table 2:** The physical properties of polymers.

Parameters	Specifications	HDPE	LDPE
Density (g/cm^3^)	ASTM D 792	0.037	0.033
Absorption (%)	ASTM D 570	0	<0.01
Tensile Strength (psi)	ASTM D 638	4400	1900
Yield (%)	ASTM D 638	900	710
Melting Temp. (°C)	ASTM D 3418	125	110
Flexural modulus (psi)	ASTM D 790	20,000	-

**Table 3:** Low- and high-performance grade testing of HDPE and LDPE modified bitumen.

Bitumen + LDPE	Bitumen + HDPE	High		Low	PG
		Performance			
		Original	RTFO *	BBR	
ARL + 0% LDPE	ARL + 0% HDPE	58	58	−12	58–22
ARL + 2% LPDE	ARL + 2% HPDE	64	64	−12	64–22
ARL + 4% LDPE	ARL + 4% HDPE	70	70	−12	70–22
ARL + 6% LDPE	ARL + 6% HDPE	76	70	−12	70–22

* Rolling Thin Film Oven (RTFO), Bending Beam Rheometer (BBR), Attock Refinery Limited (ARL), High-Density Polyethylene (HDPE). Low-Density Polyethylene (LDPE).

**Table 4:** RTFO of HDPE and LDPE modified bitumen (PG 58-22).

Bitumen + Polymer	Temperature (°C)	Phase Angle (θ)	G*/Sin δ (kPa)
PG + 0%	25	55.32	4000
PG + 2% HDPE	25	54.20	5900
PG + 4% HDPE	25	51.12	7503
PG +6% HDPE	25	49.59	11,200
PG + 2% LDPE	25	53.88	4700
PG + 4% LDPE	25	52.47	5620
PG +6% LDPE	25	54.97	9700

**Table 5:** RTFO of HDPE and LDPE modified bitumen (PG 58-22).

Bitumen + Polymer	Temp (°C)	Phase Angle (θ)	G*/Sinδ (kPa)
PG + 0%	58	86.28	2000
PG + 2% HDPE	58	85.23	6203
PG + 4% HDPE	58	83.28	7500
PG + 6% HDPE	58	82.21	3200
PG + 2% LDPE	58	84.70	2400
PG + 4% LDPE	58	85.78	1550
PG + 6% LDPE	58	85.90	1950

## Data Availability

All the necessary data required for supporting this research work are included in this paper.
